# From Single Batch to Mass Production–Automated Platform Design Concept for a Phase II Clinical Trial Tissue Engineered Cartilage Product

**DOI:** 10.3389/fmed.2021.712917

**Published:** 2021-08-13

**Authors:** Sebastian Haeusner, Laura Herbst, Patrick Bittorf, Thomas Schwarz, Chris Henze, Marc Mauermann, Jelena Ochs, Robert Schmitt, Ulrich Blache, Anke Wixmerten, Sylvie Miot, Ivan Martin, Oliver Pullig

**Affiliations:** ^1^Translational Center Regenerative Therapies TLC-RT, Fraunhofer Institute for Silicate Research, Wuerzburg, Germany; ^2^Department of Tissue Engineering and Regenerative Medicine, University Hospital Wuerzburg, Wuerzburg, Germany; ^3^Fraunhofer Institute for Production Technology IPT, Aachen, Germany; ^4^Fraunhofer Institute for Process Engineering and Packaging IVV, Dresden, Germany; ^5^Laboratory for Machine Tools and Production Engineering (WZL), RWTH Aachen University, Aachen, Germany; ^6^Fraunhofer Institute for Cell Therapy and Immunology, Leipzig, Germany; ^7^Department of Biomedicine, University Hospital Basel, University of Basel, Basel, Switzerland

**Keywords:** ATMP, tissue engineering, GMP, manufacturing, autologous, cartilage regeneration, automation & robotics, automation

## Abstract

Advanced Therapy Medicinal Products (ATMP) provide promising treatment options particularly for unmet clinical needs, such as progressive and chronic diseases where currently no satisfying treatment exists. Especially from the ATMP subclass of Tissue Engineered Products (TEPs), only a few have yet been translated from an academic setting to clinic and beyond. A reason for low numbers of TEPs in current clinical trials and one main key hurdle for TEPs is the cost and labor-intensive manufacturing process. Manual production steps require experienced personnel, are challenging to standardize and to scale up. Automated manufacturing has the potential to overcome these challenges, toward an increasing cost-effectiveness. One major obstacle for automation is the control and risk prevention of cross contaminations, especially when handling parallel production lines of different patient material. These critical steps necessitate validated effective and efficient cleaning procedures in an automated system. In this perspective, possible technologies, concepts and solutions to existing ATMP manufacturing hurdles are discussed on the example of a late clinical phase II trial TEP. In compliance to Good Manufacturing Practice (GMP) guidelines, we propose a dual arm robot based isolator approach. Our novel concept enables complete process automation for adherent cell culture, and the translation of all manual process steps with standard laboratory equipment. Moreover, we discuss novel solutions for automated cleaning, without the need for human intervention. Consequently, our automation concept offers the unique chance to scale up production while becoming more cost-effective, which will ultimately increase TEP availability to a broader number of patients.

## Introduction

ATMPs are at the forefront of current state of the art medical science and technology. This innovative and complex class of biological products promises new therapeutic options for yet unmet medical needs. Currently, only 12 ATMPs hold European marketing authorization, mostly composed of Gene Therapy Medicinal Products (GTMP) and with merely two TEPs according to regulation EC1394/2007 (1, 2). Chronic and progressive diseases still pose major clinical challenges for conventional and even advanced therapies, especially for tissues with only limited regenerative capacity. Untreated injuries of articular cartilage for example may lead to progressive loss of cartilaginous as well as osseous tissue because of its limited ability for self-repair. Annually about two million people are affected by cartilage injuries in Europe and the United States alone, with significant effects on the patients' quality of life due to severe pain and impaired function, particularly in the joints (3). Moreover, if left untreated, these lesions predispose to the onset of osteoarthritis, which might ultimately necessitate total joint replacement. Even though results published for joint arthroplasty are generally satisfactory, 10–15% of the patients are dissatisfied and report complications (4). Furthermore, especially in younger patients (<60 years) the risk of revision surgery, associated with lower treatment efficacy, is increased by 20–35% in total (5, 6). Current treatments for focal cartilage defects, e.g., microfracturing or autologous articular chondrocyte implantation, are often associated with drawbacks such as limited defect sizes and donor site morbidity (7–9). Various new treatment approaches using ATMPs are currently under scientific investigation, also in clinical trials with mainly somatic cell therapy medicinal products (sCTMP) among others (10, 11).

## Manual ATMP Manufacturing and Limitations

A tissue engineering approach using autologous nasal septum derived cartilage cells, cultured on a 3D carrier matrix for treatment of focal cartilage defects reveals promising outcomes in a phase I (clinicaltrials.gov identifier: NCT01605201) and ongoing phase II clinical trial (clinicaltrials.gov identifier: NCT02673905) (12–14). These nasal chondrocyte tissue engineered cartilages (N-TECs) are combined ATMPs, consisting of autologous nasal chondrocytes, cultured on a certified, commercially available collagen membrane (Chondro-Gide®, Geistlich Biomaterials), and extracellular matrix produced by the cells.

**Figure 1:**
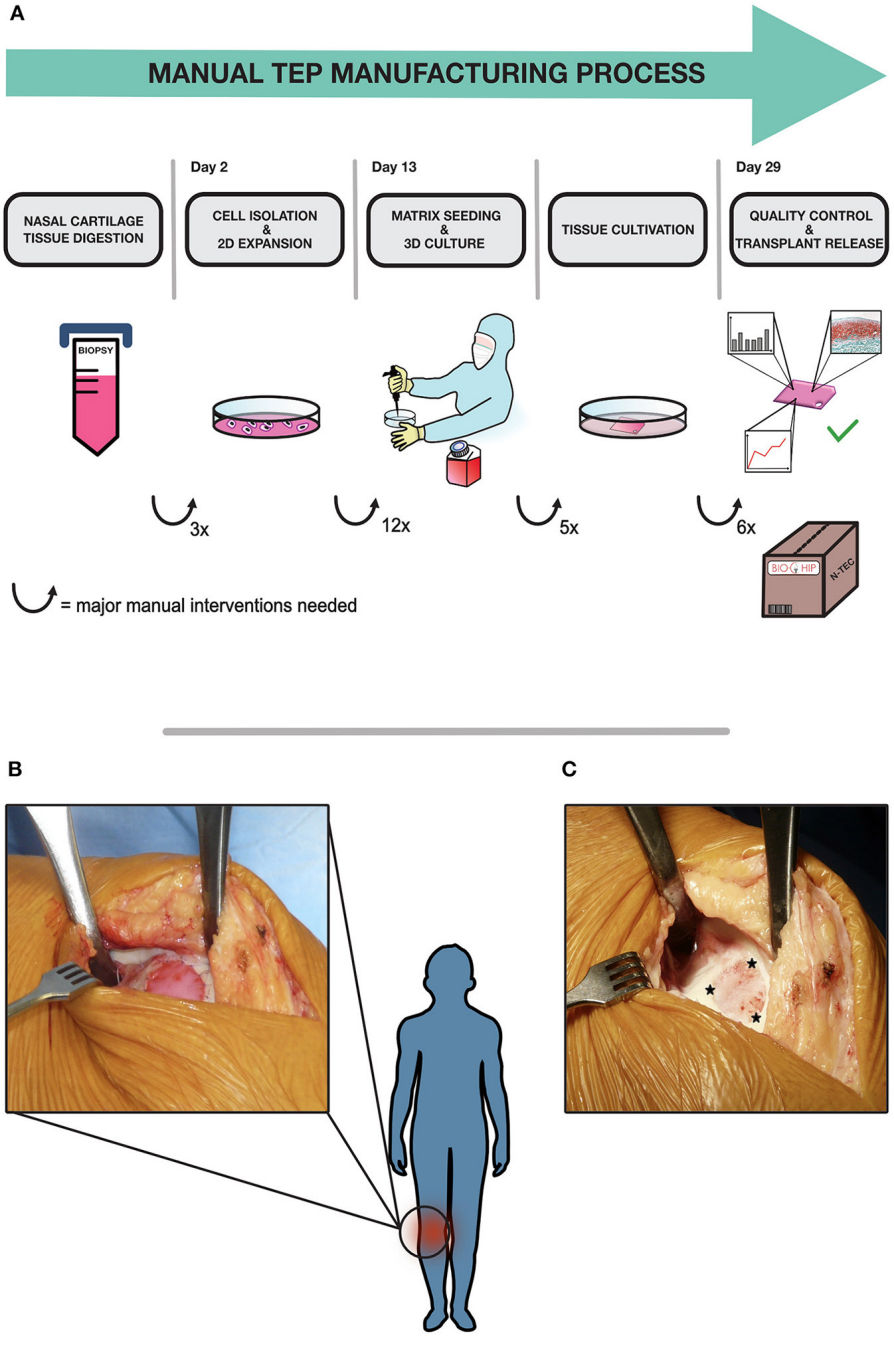
BIO-CHIP manual manufacturing process and surgical procedure. The whole process is graphically depicted in **(A)**. An autologous cartilage biopsy is taken via outpatient surgery in a clinical site from the patients' nasal septum according to SOP. The biopsy is shipped to the manufacturing site, where the tissue is digested and the cartilage cells are isolated and expanded *in-vitro*. In the next step, cells are manually seeded on a collagen membrane in a certified cleanroom facility. Various parameters are monitored continuously throughout tissue cultivation. After 2 weeks of static tissue culture, final quality testing is conducted. The amount of extracellular matrix proteins is evaluated using histological grading by modified Bern score, also cell viability and transplant stability are assessed. When all defined release criteria are met, the N-TEC is packed and sent back to the clinic to be implanted into defect site in a surgical procedure **(B)**. The patch is secured by surrounding absorbable sutures during the surgical procedure. The initial focal cartilage defect in the knee is depicted in **(C)**, asterisks indicate the defect site where the N-TEC is inserted.

The combined ATMPs are manufactured in a manual way, graphically depicted in Figure 1A, in a cleanroom facility according to GMP guidelines and Standard Operating Procedures (SOP). The demanding process relies on authorized manufacturing sites, highly trained personnel and rigorous quality controls in order to ensure continuous high product quality. In the first step a small cartilage biopsy is obtained from patient's nasal septum in an authorized clinical site. The biopsy is shipped to the manufacturing site by a validated transport procedure. Upon arrival, the cartilage sample is minced and chondrocytes are released from the biopsy by enzymatic digestion. The cells are expanded for 13 days in 2D *in-vitro* culture, which require daily medium changes for the first 3 days in the first expansion phase and medium changes every 3 days in the second expansion phase, including manual sampling for cell counting and microbial testing at the end of the expansion. Cells are passaged two times with four T175 cm^2^ cell culture flasks required for each product, as described elsewhere (14). Once sufficient cell numbers are available, they are manually seeded at a defined density onto the scaffold to generate a 3D tissue in static culture. After a total culture period of 29 days, the TEP is tested and shipped to the clinical site and surgically implanted into the focal cartilage lesion (Figure 1B), to promote cartilage regeneration and mitigate disease onset (Figure 1C). All manual handling steps, such as biopsy mincing, media changes, cell seeding and sampling for in-process-controls and release tests, are currently conducted in an EU-GMP grade A (United States Food and Drug Administration (FDA)-Class 100) safety cabinet inside a grade B environment under laminar air flow. Despite highly qualified personnel, strict adherence to SOPs and an efficient process, current manual manufacturing is afflicted with several disadvantages regarding costs, scale up, reproducibility and standardization. The main cost drivers in the process are salary costs for qualified personnel during the labor-intensive process, as well as the operational costs for running a GMP cleanroom-facility. Although some cost reduction could be achieved by parallelization, there are clear limits to the upscaling of the process due to working hour restrictions and limited cleanroom capacity, additionally to an expectable shortage of qualified personnel. Another drawback in the manual manufacturing process is the limitation in terms of standardization and thus reproducibility. Critical steps like the distribution of cell suspension on the matrix surface requires extensive training, skills and experience to ensure homogeneity in cell distribution and equal matrix production throughout the scaffold. These particular steps are subject to inter- and intra-operator variability, additionally to the intrinsic variability of an autologous biological product and donor-batch-variations (15–17). This lack of standardization may affect the quality and reproducibility of the final product and also impedes the transferability of processes to other manufacturing centers. Moreover, the current open manufacturing system, the frequent manual process steps as well as the invasive final quality control testing, pose major risks for contaminations throughout the process chain. Overcoming these hurdles is a prerequisite to achieve a more time- and cost-effective, standardized and scalable automated manufacturing process.

## Automated ATMP Manufacturing Concept

**Figure 2:**
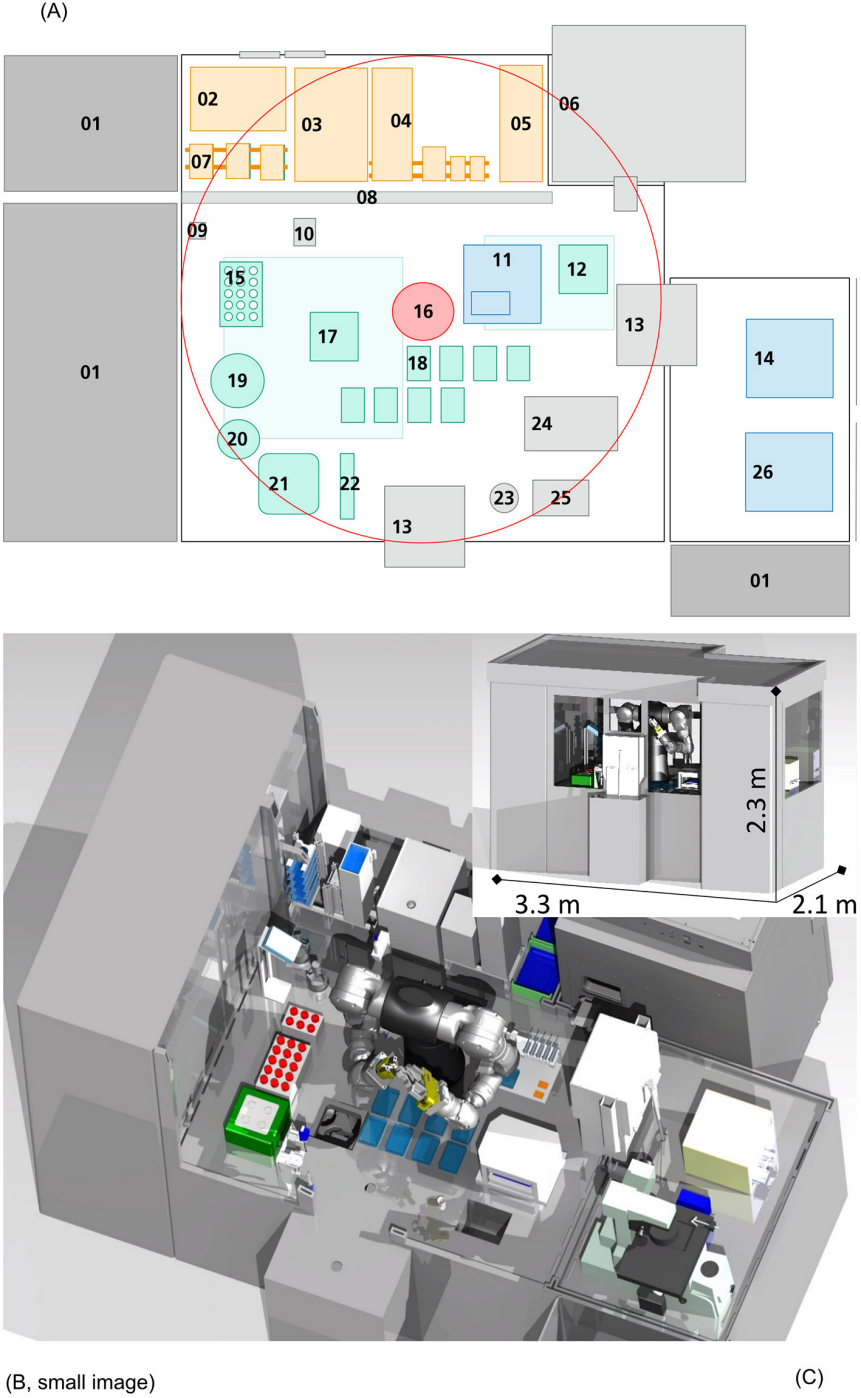
Automation scheme and visual representation. In this figure, a 2D **(A)** and 3D [**(B)**: frontal view, **(C)**: sliced view] representation of this concept is shown. The platform is equipped with devices for each handling step: 01 Ventilation system, 02 Freezer (−20°C), 03 Fridge (4°C), 04 Disposables storage, 05 Packaging material, 06 Incubator (37°C), 07 Storage for plates and membranes, 08 Gate, 09 Barcode reader, 10 Washing station, 11 Sampling station, 12 Shaker, 13 Air-lock, 14 Cell counting device, 15 Storage for Cell culture tubes (temperature controlled), 16 Six axis dual arm robot, 17 Centrifuge, 18 Plate handling positions, 19 Decapper (centrifuge flask), 20 Decapper (Cell culture tubes), 21 Tissue grinder, 22 Pipettes, 23 Liquid waste, 24 Sealing machine, 25 Solid waste, 26 Microscope. The central six axis dual arm robot (16) can reach the circumference shown in **(A)**. Necessary equipment, disposables and liquids are safely channeled in through an air lock linked glove box (orange), without the need of personnel entering the isolator directly from an unclassified maintenance back side. Pre-packed, sanitized disposables, materials or biologicals can be unpacked easily and set in place for robot-driven procession. Devices for cell culture (green) and quality control (blue) are included in the design. The platform is scaled to the parallel production of five N-TECs at a time, with the possibility of increasing manufacturing capacity further e.g., by implementation of larger storage devices.

Automation of ATMP manufacturing processes is a key technology to bring these translational pathways from bench to late phase III clinical trials and beyond. This perspective presents a concept for automation of the N-TEC process with the potential for a higher, more cost-efficient manufacturing capacity, process standardization and facilitated regulatory-compliant in-process documentation. The evolving field of ATMP manufacturing, especially during early process developments, necessitates a certain flexibility for such an automation concept. Although there are already many applications and disposable bioreactors available for suspension cell culture (18, 19), only very limited options exist for automated adherent cell culture (20). Moreover, even the production of sCTMPs like CAR-T or natural killer cells mainly relies on partly automated suspension culture systems for clinical scale manufacturing (21–26). Additionally, the few commercially available semi-automated solutions for adherent cell culture systems require special single-use disposables additionally to high acquisition costs. Efforts on the part of the scientific community toward automated adherent cell production are currently investigated, with novel automated modular approaches for human mesenchymal stem cells by Kikuchi et al., or either commercially available products, like the description of the first adherent cell culture using CliniMACS Prodigy by Vieira et al. (27, 28). However, a fully automated platform that is highly adaptable to various specific steps, such as cell isolation involving mincing, or handling of cells from different donors in parallel without facing cross contamination issues, has not been described before. We propose a more adjustable design that is suitable for suspension cell culture as well as plate-based approaches, including adherent cell and tissue culture in a validated platform. The automated two arm robot design concept enables complete process automation of all manual process steps with standard laboratory equipment. Combined with necessary handling and storage units, quality control and regulatory framework in mind, the isolator is equipped with devices typically used in a tissue engineering facility, as shown in Figure 2. Our concept is also designed for automated cleaning procedures, a unique feature not addressed by other automated culture systems. To best of our knowledge, no fully automated manufacturing system is currently in use in academia nor other manufacturers of TEPs.

## The Design of Automation

The very center of the conception is based on a dual arm, six axis robot unit [Figure 2A (16), red]. Due to its high degree of freedom in movement, it allows for the implementation of complex tasks, and “human-like” robotic operations as liquid handling and cell culture (Figures 2B,C). Hence, manual protocols, previously developed in the lab, could be easily translated to be carried out by the robot as independent processes, without major alterations in equipment or process steps. The storage area (orange) is accommodated from an unclassified maintenance back side by linked glove box handling, without personnel entering the isolator directly. Pre-packed, sanitized disposables and materials can be unpacked easily and set in place for robot-driven procession. The concept follows a GMP-compliant unidirectional workflow, where all necessary disposables, liquids and starting materials (patient samples) enter the isolator through an air lock system and leave the aseptic environment through another air lock as final product. Patient samples are processed at a tissue culture area (green), where tissue mincing/digestion, cell isolation, seeding and tissue culture is conducted. Each handling step and consumable used in the process can be traceable through barcode-based recognition (09), ensuring continuous facilitated LOT-specific digital documentation and thus automating documentation. This automated continuous in-process documentation can significantly reduce mandatory regulatory paperwork, avoid sample mix ups and enhance product traceability as well as process transparency, eliminating time consuming manual protocol writing. On the direct opposite side, the sampling station (11) is located. This setup facilitates sampling during culture medium changes and further reduces handling distances of liquids within the operating plant. Samples are directly channeled through air locks to an adjoining quality control area (blue) [Figure 2A, (14,26)]. Trained members of the quality assurance unit accept the samples for manual inspection and testing. In this concept quality control of ATMPs is still conducted by experienced personnel, as the focus is on automating all steps involved in he N-TEC manufacturing. Ventilation systems (01) filter air through High Efficiency Particulate Air filter units (HEPA) and establish aseptic environmental conditions with only minimal amounts of airborne particle collectives. Vast incubator units (06) monitor and control humidity, air flow, CO_2_-levels and temperature with storage space for cell culture plates for up to five TEPs at a time in parallel. Current manufacturing is heavily reliant on trained lab personnel whose interventions are also considered to be the main source of contamination in the aseptic manufacturing environment concerning FDA and European Medicines Agency (EMA). Implementing an isolator-type platform, allows for the separation of product and manufacturer, thus limiting human interaction and greatly reducing the risk of contamination. However, intricate cleansing by hand between production campaigns, clearly scotches these benefits nonetheless. Especially the performance of sequential manufacturing operations, with different patient material necessitates the development of quick and efficient cleaning procedures in between process steps and variant batch productions, to avoid cross contaminations according to EudraLex IV Point 4.26 (29). We propose to include automated cleaning procedures for all automated parallelized manufacturing systems of ATMPs. Current cleaning methods include gassing with hydrogen peroxide and a subsequent wipe-down or using other decontamination reagents during wipe-down. While gassing and wipe-down are thought to be possible to be implemented in an automated ATMP production plant, it is estimated that the whole procedure would take too long to be performed in between process steps handling cell material from different patients. To avoid cross contamination and accelerate the cleaning process, an approach based on spray nozzles is highly suggested. In similar approaches from the food industry, decontamination and cleaning reagents would be sprayed across surfaces and devices to remove any potential residual cell material. The platform is further equipped with a spray nozzle that may be used by the dual arm robot to clean any unsprayed areas [2A, (10)]. Afterwards the surfaces are dried with sterilized compressed air. Implementing such Cleaning-In-Place and Sterilization-In-Place based concept has great implications for the design of the production platform, as it has to be sealed off and proper drainage has to be achieved. As not all devices are suitable for such a procedure, the platform is compartmentalized into different modules according to their necessary functions. As of today, and to the best of our knowledge, such a GMP conform cleaning procedure has not yet been tested for automated ATMP manufacturing platforms and needs to be evaluated toward its efficacy and regulatory compliance.

## Standardization For a More Reliable, Autonomous Production

Another challenge of the current manual production is the potential inter- and intra-operator variability during seeding of the scaffold and to a lesser extent the cartilage digestion. Thus, automation of these steps has great potential, in particular to reduce variations due to operator handling in the final product. In the proposed automation concept, cell seeding is performed by the dual arm six axis robot. However, the complexity of the cell seeding process will require careful implementation and meticulous testing before standardization by automation of this step is achieved. In the long term, standardization of this process step would reduce the process-induced variability and allow for a more reproducible and high-quality production. In general, the most complex steps are the ones most relevant for automation. But also automation of time consuming repetitive steps, like manual medium exchange, could quickly impact on cost-effectiveness. Moreover, the risk of human error is reduced to an absolute minimum when handling production in a closed automated system in contrast to open manual handling.

## Automation and Cost-Effectiveness

Especially with constantly rising personnel wages, an automated production will become even more cost-effective from an economic perspective. In the complex production of iPS cells, personnel costs account for nearly 60% of total manufacturing costs, with estimably 42% more manual production costs than in automated production (30). These costs are comparable to a sophisticated TEP product like the N-TEC, which ranges from 17,000–20,000 € per product with frequent manual interventions and produced in an academic setting. The investment for automated iPS production is estimated to be about 1,000,000 €, whereas the investment for the automated TEP production plant in total is estimated 1,500,000 €, with additional operational resources as described elsewhere (30). Even though the initial payback period a TEP-facility might also be longer, than those of a cell production line, with an increasing TEP market availability, positive cash flow could be achievable within the first 3 years of market acceptance. Parallelization could further lift single-cost burdens, and greatly benefit to a fast scale up process. Additionally, an automated production platform is independent of working hour restrictions and can be designed to a very confined space, superseding cost-intensive walk-in cleanroom structures.

## ATMP Manufacturing 4.0: Facilities of the Future

The main objective of this work is the mere automation of a former manual process with a rather deterministic robotic unit. This transitional step could pave the way for future technology and applications that will further improve the production process. Our current ATMP manufacturing protocol and the concept for the automated platform incorporates invasive sampling procedures prior to product release and manual scoring to ensure high product standards to pre-defined quality criteria. Although not yet integrated in the concept, first approaches to automate these controls have already been carried out. As an example, the automated visual inspection of histological tissue engineered cartilage using a modified Bern score and deep learning algorithm has already been demonstrated to be a feasible method for the prospective evaluation and graft release in a clinical manufacturing setting (31, 32). However, these pivotal procedures would greatly benefit from the implementation of non-invasive in-process controls, enabling real-time quality control and monitoring of product specifications throughout the manufacturing process. Appropriate methods using non-invasive sampling e.g., supernatant for cell viability determination through lactate dehydrogenase assay, are currently reviewed and validated for GMP compliant manual application. Other possible technologies for such a non-invasive quality control would include optical coherence tomography (OCT) or Raman spectroscopy, which are currently under evaluation regarding quality controls for TEPs prior to implementation in the process (33). The ultimate goal is to monitor and collect high quality product related data (e.g., cell population doubling, temperature, oxygen, pH) through equipped sensors in order to employ data driven approaches for process and schedule optimization. Non-invasive continuous monitoring along with model-based strategies have the potential to supersede current invasive quality monitoring and to enable a true “smart factory” setting. *In-silico* tools employing artificial intelligence to predict process outcome might be used to predict the best harvesting time points, before cells have reached critical mass (34). This would be highly beneficial for the quality and outcome of *in-vitro* generated tissues by accounting for the individual needs and nature of patient derived cells, but also facilitate the critical release testing. With product data sets from continuous monitoring, *in-silico-predictions* could finally allow a near real-time release of TEPs matching individually scheduled dates for surgical procedures, without the risk of delay due to product inconsistencies.

## Discussion

Many ATMPs are currently under review and in preparation for at least partly automated manufacturing systems, on the market as well as in research and clinical facilities. But we also observe from experience, that the latter is introduced far beyond the point of product development, in late stages of testing which makes it difficult to develop a platform that is adaptable to all needs of the product manufacturing. We propose to keep the concept of automation always in mind when initially developing a manufacturing process to ease initial implementation, and to avoid unnecessary expenses for product revalidation (35). Dual-use equipment for example, which is suitable for manual laboratory work, but also eligible to be implemented in an automated setting, would prevent doubling the high acquisition costs for a manual and automated process later on. The question how far a biological process can really be improved above the limits of inherent biological variance is a tough one. It can only be addressed properly, when such an automated manufacturing system has actually been built and successfully been tested in full extent. Currently, automation concepts are hindered by comparatively small technical pitfalls, such as the lack of automated cleaning procedures. However, they also add on the whole new topic of validating of the automation software and procedures according to good automated manufacturing practice (GAMP). Several automated platforms and devices have been developed for cell culture and ATMP production, however these are either reliant on disposable inserts or hazardous manual cleaning procedures that necessitate an interruption between production campaigns. Automated manufacturing becomes more cost-effective especially when parallelizing production to a high extent. To avoid long down-times before, and cross contamination during production, automated cleaning procedures must be developed and validated for these platforms. Moreover, technical challenges in facility design for these cleaning procedures must be overcome, wherefore more research and development in that area is needed. But is it worth the effort in the end? Early cost-intensive investments in automation may seem irrational at first, but will pay off in form of a consistent process for phase I, and more scalable product for phase II/III clinical trial stages. We conclude that implementing 4.0 standards and new manufacturing methods to TEP and ATMP production in general must be more than just a scientific exercise. Automation will help to unleash the full economic potential of ATMPs in an ever competitive drug market. Only then, with a positive cost-benefit-ratio, living drugs will be appealing to health care providers and insurances. This will ultimately help to deliver more ATMP based therapies to patients in dire need.

## Data Availability Statement

The raw data supporting the conclusions of this article will be made available by the authors, without undue reservation.

## Author Contributions

SH wrote the first draft of the manuscript. LH and SH created the figures. SH, LH, SM, and AW wrote sections of the manuscript. All authors contributed to the conception and design of the perspective, contributed to manuscript revision, read, and approved the submitted version.

## Conflict of Interest

The authors declare that the research was conducted in the absence of any commercial or financial relationships that could be construed as a potential conflict of interest.

## Publisher's Note

All claims expressed in this article are solely those of the authors and do not necessarily represent those of their affiliated organizations, or those of the publisher, the editors and the reviewers. Any product that may be evaluated in this article, or claim that may be made by its manufacturer, is not guaranteed or endorsed by the publisher.
